# Complexities of pharmacogenomic interactions in cancer

**DOI:** 10.1080/23723556.2020.1735910

**Published:** 2020-03-19

**Authors:** Kum Kum Khanna, Pascal H.G. Duijf

**Affiliations:** a QIMR Berghofer Medical Research Institute, Herston, Australia; b Institute of Health and Biomedical Innovation, School of Biomedical Sciences, Faculty of Health, Queensland University of Technology, Brisbane, Australia; c Translational Research Institute, University of Queensland Diamantina Institute, the University of Queensland, Brisbane, Australia

**Keywords:** Cancer, pharmacogenomics, genomic instability, drug response, drug sensitivity, drug resistance, precision medicine

## Abstract

Genetic and genomic alterations drive cancer development. However, they may also constitute vulnerabilities, including increased drug sensitivity, which could be harnessed for precision medicine purposes. We discuss the highly complex pharmacogenomic interactions that are beginning to be disentangled and hurdles that may need to be overcome before cancer patients could benefit.

## Main text

As they develop, cancers acquire genetic and genomic abnormalities, a phenomenon referred to as genomic instability. A range of underlying molecular mechanisms have been identified, such as defects in the DNA damage checkpoint, DNA damage repair, DNA replication, mitotic checkpoint signaling and cytokinesis.^1^ The mechanisms and consequences of genomic instability, including mutations, focal copy number alterations (CNAs), aneuploidy and translocations, may offer opportunities for the treatment of cancer patients.

The field of cancer pharmacogenomics aims to identify interactions between cancer genomic alterations and drug response.^2^ It has a long history with a number of remarkable successes. The Philadelphia chromosome in chronic myeloid leukemia (CML) patients involves a reciprocal 9q:22q translocation. This generates the *BCR-ABL1* fusion gene (between the *Breakpoint Cluster Region* and *Abelson Tyrosine-Protein Kinase 1* genes), whose expression sensitizes to the kinase inhibitor imatinib (Gleevec).^3^ Similarly, oncogenic mutations or focal amplifications of the loci encoding the epidermal growth factor receptors EGFR (Epidermal Growth Factor Receptor) and HER2 (Human Epidermal Growth Factor Receptor 2) are common in lung and breast cancers and increase sensitivity to cognate inhibitors, including gefitinib, erlotinib, cetuximab, trastuzumab and lapatinib.^2^ However, how cancers respond to these agents is influenced by individual tumor genetic contexts. *HER2*-amplified tumors with loss of *PTEN* (*Phosphatase And Tensin Homolog*) do not respond to trastuzumab^4^ and *EGFR*-amplified non-small cell lung and colorectal cancers with mutations in *KRAS* (*Kirsten rat sarcoma viral proto-oncogene*) do not respond to EGFR-targeted agents.^4^

In 2016, numerous new pharmacogenomic interactions were identified using cancer cell lines derived from 18 cancer types.^5^ These and above examples mostly comprise ‘simple’ pharmacogenomic interactions, as they involve mutations, focal CNAs or translocations primarily involving single (fusion) genes. However, more complex interactions exist too. For example, said study also identified combinations of genomic alterations that alter drug response.^5^

Poly-ADP ribose polymerase (PARP) inhibitors represent another example. Their clinical benefit for the treatment of various cancers, in particular breast and ovarian cancers with mutations in *BRCA1* or *BRCA2* (breast cancer type 1 and 2 susceptibility proteins) largely relies on a principle called ‘synthetic lethality,’ a concept in which defects in one gene have minimal effects on cells, but defects in a combination of genes are cell-lethal.^6^ PARP inhibitors trap PARP onto the DNA and subsequently cause DNA replication-associated DNA double-strand breaks. In normal cells, these can be repaired via homologous recombination repair (HRR). However, HRR-defective cancer cells, including cells with *BRCA1*/*2* mutations, cannot repair this damage and die.^7^ Thus, although drug resistance may arise, PARP inhibitors can selectively eradicate HRR-deficient cancer cells.

Recently, we utilized an expanded version of the extensive pharmacogenomic cell line resource referred to above^5^ (cancerrxgene.org) to assess whether additional complex forms of cancer pharmacogenomic interactions exist.^8^ Chromosome arm aneuploidies (CAAs) are common in human tumors and on average affect 25% of the cancer genome, compared to focal CNAs affecting 10%.^9^ Thus, we reasoned that simultaneous copy number gains or losses of genes encoded on chromosome arms, due to CAAs, could comprise a novel form of complex pharmacogenomic interactions.

**Figure 1.**
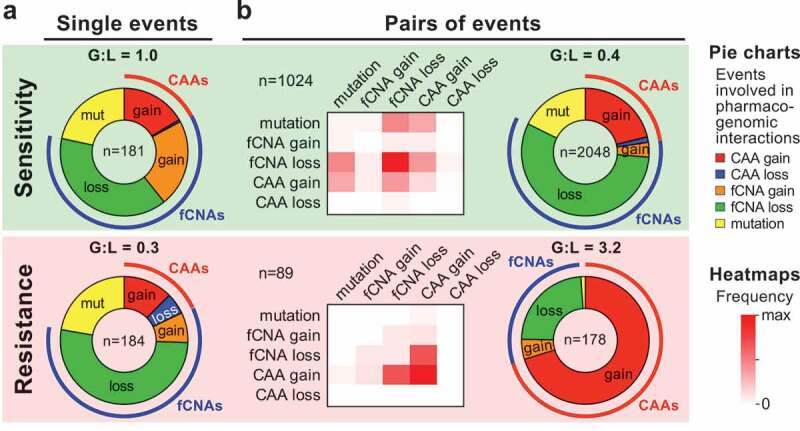
Complex pharmacogenomic interactions in cancer cells. Meta-analysis of recently identified pharmacogenomic interactions in cancer cell lines,^8^ including mutations (mut), focal copy number alterations (fCNAs) and chromosome arm aneuploidies (CAAs) – the latter two including gains and losses. Pie charts show the distributions of interactions involving single genomic events (a) and pairs of co-occurring genomic events (b). Ratios of events involving gain and loss (G:L) are shown above each pie chart. Heatmaps show the frequencies of co-occurring events involved in drug interactions. Events associated with increased drug sensitivity or resistance are shown against green and red backgrounds, respectively. Source data are in Suppl. Data 10 and 11 of reference ^8^.

Indeed, an unbiased machine learning approach that included both well-established cancer functional events (CFEs, i.e., common cancer gene mutations and focal CNAs; n = 710) and CAAs (n = 78), as well as IC_50_ values (half of the maximum inhibitory concentration of a drug) pertaining to 453 drugs and 988 cell lines, identified 365 robust CFE- or CAA-drug interactions.^8^ This involved approximately equal numbers of drug sensitivity (n = 181) and resistance interactions (n = 184) (Figure 1a). However, the number of events involving copy number loss is considerably higher for those involved in drug resistance. Importantly, this includes ‘simple’ interactions, with drug resistance linked to copy number loss of the drug target gene, as well as ‘complex’ interactions. For instance, of all 64 identified CAA-drug interactions, only two can be explained by focal CNAs or any combination of two focal CNAs affecting the same chromosome arm.^8^ Thus, CAAs represent a new form of complex cancer pharmacogenomic interactions.

We also assessed potential associations between drug response and pairs of any two genomic events, as these could uncover potential synthetic lethal or synergistic drug resistance interactions. Altogether, we identified 1024 and 89 of such interactions, respectively.^8^ Meta-analysis of these shows that focal copy number loss and chromosome arm gains dominate involvement in such sensitivity and resistance interactions, respectively (Figure 1b). Notably, the ratios between events involving copy number gain and loss are radically different from single-event interactions (compare the gain:loss ratios ‘G:L’ in Figure 1a,b). Also, chromosome arm losses are rarely involved (2.6% overall), but arm gains are involved in >50% of interactions, in particular in drug resistance interactions (91% of interactions, accounting for 70% of all involved events; Figure 1b). This underscores that CAAs shape drug response. In fact, we demonstrated that CAAs considerably outperform mutations and focal CNAs in predicting drug response.^8^ Additionally, this demonstrates the complexity of some pharmacogenomic interactions in cancer cells.

A notable example of complexity is the identification of chromosome arm 17p loss and resistance to seven drugs in acute myeloid leukemia.^8^ Six of these drugs are cell cycle inhibitors and *TP53*, encoding the key cell cycle regulator TP53 (best known as p53), is located on 17p, suggesting involvement of p53. However, neither *TP53* loss nor loss of any combination of the *TP53* locus and any focal region on 17p is implicated in resistance to any of these drugs – although *TP53* mutations are linked to resistance to one of the drugs.^8^ This suggests the existence of a complex pharmacogenomic interaction implicating resistance to cell cycle inhibitors to concurrent loss of *TP53* and at least two other loci on 17p.

Finally, we emphasize that in a broader context, there are several other levels of complexity, all of which need to be considered before either ‘simple’ or ‘complex’ pharmacogenomic interactions can be translated and potentially provide therapeutic value for cancer patients. First, cancer cells often harbor other alterations in addition to those mentioned above, including complex rearrangements (resulting from chromothripsis or breakage-fusion-bridge cycles), double-minutes, fusion genes, whole-genome doubling and epigenetic alterations.^10^

Second, the existence of intra-tumor heterogeneity, with sub-clonal genomic differences between cells within the same tumor, may complicate the efficacy of a treatment based on pharmacogenomic interactions. However, targeting ‘truncal’ or clonal alterations common in all tumor cells may overcome this.

Third, cancer cells may acquire drug resistance, which is also observed with aforementioned treatments involving translocations in CML, *EGFR/HER2* mutations/amplifications and PARP inhibitors.^2,3,7^

Fourth, the context of the tumor microenvironment needs to be considered, as the levels of infiltrating immune cells, fibroblasts and vasculature may affect drug efficacy.

Fifth, variation of the patient’s germline may influence both drug response and tolerance, potentially causing adverse side effects or toxicity.

Lastly, safety, dosing and efficacy of drugs in relation to new pharmacogenomic interactions may first require testing in cost- and time-intensive pre-clinical experiments and clinical trials.

Taken together, a range of cancer pharmacogenomic interactions have recently been identified. The nature of most complex interactions remains poorly understood and a number of hurdles need to be overcome before cancer patients could benefit. However, we are in an exciting era in which new cancer pharmacogenomic interactions are identified at an accelerated pace, offering hope for clinical implementation in the not too distant future.
